# Vitamin D (1α,25(OH)_2_D_3_) supplementation minimized multinucleated giant cells formation and inflammatory response during *Burkholderia pseudomallei* infection in human lung epithelial cells

**DOI:** 10.1371/journal.pone.0280944

**Published:** 2023-02-09

**Authors:** Pohnratchada Mattrasongkram, Saharut Wongkaewkhiaw, Suwimol Taweechaisupapong, Sorujsiri Chareonsudjai, Teerasit Techawiwattanaboon, Thararin Ngamsiri, Sakawrat Kanthawong

**Affiliations:** 1 Department of Microbiology, Faculty of Medicine, Khon Kaen University, Khon Kaen, Thailand; 2 School of Dentistry, King Mongkut’s Institute of Technology Ladkrabang, Bangkok, Thailand; 3 Department of Oral Biomedical Science, Faculty of Dentistry, Khon Kaen University, Khon Kaen, Thailand; 4 Research and Diagnostic Center for Emerging Infectious Diseases (RCEID), Khon Kaen University, Khon Kaen, Thailand; 5 Department of Microbiology, Faculty of Medicine, Chulalongkorn University, Bangkok, Thailand; 6 Chula Vaccine Research Center (Chula VRC), Center of Excellence in Vaccine Research and Development, Chulalongkorn University, Bangkok, Thailand; Babasaheb Bhimrao Ambedkar University, INDIA

## Abstract

Melioidosis is an infectious disease with high mortality rates in human, caused by the bacterium *Burkholderia pseudomallei*. As an intracellular pathogen, *B*. *pseudomallei* can escape from the phagosome and induce multinucleated giant cells (MNGCs) formation resulting in antibiotic resistance and immune evasion. A novel strategy to modulate host response against *B*. *pseudomallei* pathogenesis is required. In this study, an active metabolite of vitamin D_3_ (1α,25-dihydroxyvitamin D_3_ or 1α,25(OH)_2_D_3_) was selected to interrupt pathogenesis of *B*. *pseudomallei* in a human lung epithelium cell line, A549. The results demonstrated that pretreatment with 10^−6^ M 1α,25(OH)_2_D_3_ could reduce *B*. *pseudomallei* internalization to A549 cells at 4 h post infection (*P* < 0.05). Interestingly, the presence of 1α,25(OH)_2_D_3_ gradually reduced MNGC formation at 8, 10 and 12 h compared to that of the untreated cells (*P* < 0.05). Furthermore, pretreatment with 10^−6^ M 1α,25(OH)_2_D_3_ considerably increased hCAP-18/LL-37 mRNA expression (*P* < 0.001). Additionally, pro-inflammatory cytokines, including MIF, PAI-1, IL-18, CXCL1, CXCL12 and IL-8, were statistically decreased (*P* < 0.05) in 10^−6^ M 1α,25(OH)_2_D_3_-pretreated A549 cells by 12 h post-infection. Taken together, this study indicates that pretreatment with 10^−6^ M 1α,25(OH)_2_D_3_ has the potential to reduce the internalization of *B*. *pseudomallei* into host cells, decrease MNGC formation and modulate host response during *B*. *pseudomallei* infection by minimizing the excessive inflammatory response. Therefore, 1α,25(OH)_2_D_3_ supplement may provide an effective supportive treatment for melioidosis patients to combat *B*. *pseudomallei* infection and reduce inflammation in these patients.

## Introduction

Melioidosis is an infectious disease with high mortality rates in humans. It is caused by a Gram-negative bacterium, *Burkholderia pseudomallei* [1]. In Thailand, the first case reports appeared in 1955 and described high incidence rates in Ubon Ratchathani Province [2]. An important risk factor was contact with contaminated soil and water (through skin abrasions/wounds), such as by rice farmers during the rainy season [3]. The majority of patients with melioidosis present with pneumonia, abscess formation in various organs (e.g. lung, liver and spleen) and also sepsis resulting in organ dysfunction [4]. Importantly, *B*. *pseudomallei* can resist a variety of antibiotics (first- and second-generation penicillins, cephalosporins, macrolides, rifamycins, and aminoglycosides) [5]. As an intracellular pathogen, *B*. *pseudomallei* require flagella and adhesion-related virulence factors such as adhesins BoaA and BoaB for establishing a successful infection in both phagocytic and non-phagocytic cells [6, 7]. Then, the Bsa T3SS is required for internalization step and also play critical role for membrane disruption and escape from the endocytic vesicle [8]. Intracellular *B*. *pseudomallei* can replicate within cytosol and the T6SS-5 has been reported to play a critical role in cell-to-cell fusion resulting in the formation of multinucleated giant cells (MNGCs) [9–11]. MNGCs are a cellular feature of chronic inflammatory disorder found in tissue samples from melioidosis patients [12]. Nonetheless, the physiological functions of *B*. *pseudomallei*-induced MNGC formation are not clearly defined. Numbers of studies have suggested that MNGC formation may facilitate bacterial spread directly to adjacent cells [13] and escape from not only host innate and adaptive immune responses but also antibiotics [9]. Thus, a novel strategy to modulate host responses against the intracellular behavior of *B*. *pseudomallei* may provide a means to minimize the severity of this infection.

Vitamin D is a well-known essential micronutrient that has a classic role in the maintenance of bone mineral density [4] and a “non-classic” role in the modulation of both the innate and adaptive immune responses [14]. Dietary and dermal synthesis are the two main sources of inactive vitamin D that is later converted to the active vitamin D form, 1α,25-dihydroxy vitamin D_3_ or 1α,25(OH)_2_D_3_, by enzymes in the liver and kidney. The active form binds to the intracellular vitamin-D receptor (VDR), subsequently regulating gene expression via NF-κB in multiple signaling pathways [15] such as the inflammatory cascade and the production of endogenous cathelicidin antimicrobial peptide LL-37 [16]. Several studies have reported the association of vitamin D with the prevalence and severity of infectious diseases. Vitamin D deficiency might increase severity in patients with sepsis [17] and the risk of some respiratory infections such as influenza, tuberculosis and the recent pandemic due to severe acute respiratory syndrome coronavirus 2 (SARS-CoV-2) [18]. These effects might be via the immunomodulatory effect of vitamin D on the host innate immune response that can help prevent the initial step of infection. Vitamin D has been reported to prevent influenza virus infection by up-regulating the production of antimicrobial peptides such as defensin in primary immune cells [19] and blocking viral hemagglutinin-mediated membrane fusion [20]. Moreover, vitamin D pretreatment could increase the production of the antimicrobial peptide cathelicidin in lung epithelial cells, leading to enhanced killing activity against intracellular *Mycobacterium tuberculosis* [21] and also *Pseudomonas aeruginosa* in cystic fibrosis patients [22]. Since, vitamin D supplementation is a simple and harmless method, it is reasonable to study the role of vitamin D in prevention and treatment of other respiratory infections.

The most common clinical presentation of melioidosis patients is pneumonia: severe septicemia with high levels of pro-inflammatory cytokines are common complications [23]. Moreover, intracellular *B*. *pseudomallei* is capable of survival and movement within the cytoplasm of host cells during both acute and chronic stages [10, 13]. To the best of our knowledge, the effect of vitamin D supplement against *B*. *pseudomallei* infection has never been reported. Thus, the goal of this study was to investigate the effect of supplementation using the active metabolite of vitamin D_3_ (1α,25(OH)_2_D_3_) on adhesion and internalization of *B*. *pseudomallei* pathogenesis *in vitro*. The present study showed that 1α,25(OH)_2_D_3_ supplementation could prevent *B*. *pseudomallei* internalization and consequently reduce MNGC formation in human lung epithelial cells. Moreover, pretreatment with 1α,25(OH)_2_D_3_ can enhance the production of cathelicidin antimicrobial peptide LL-37. In addition, reduction of excessive production of inflammatory cytokines, macrophage migration inhibitory factor (MIF), plasminogen activator inhibitor type 1 (PAI-1), interleukin-18 (IL-18), C-X-C motif chemokine 1 (CXCL1), C-X-C motif chemokine 12 (CXCL12) and IL-8, during *B*. *pseudomallei* infection in 1α,25(OH)_2_D_3_-pretreated cells was observed. Thus, vitamin D supplements in melioidosis patients may reduce the excessive inflammatory response that in turn could minimize the severity of melioidosis.

## Materials and methods

### Bacterial strain and growth conditions

This study, *B*. *pseudomallei* H777, a clinical isolate (from the blood of a patient admitted at Srinagarind Hospital, Faculty of Medicine, Khon Kaen University, Khon Kaen, Thailand) [24], was selected. The single colony of *B*. *pseudomallei* H777 was inoculated into Luria–Bertani (LB) broth (Himedia^®^, Mumbai, India) and incubated aerobically at 37°C with shaking at 200 rpm overnight. To yield a mid-logarithmic growth phase, 2% inoculum of overnight culture was further cultured in LB broth at 37°C in a 200 rpm shaker-incubator for 7 h.

### Cell culture

The human lung epithelial cells (A549) (CCL-185, American Type Culture Collection, MD) were cultured in Roswell Park Memorial Institute (RPMI) 1640 Medium (Gibco™, Thermo Fisher Scientific, Grand Island, NY) supplemented with 10% (v/v) fetal bovine serum (FBS) (Gibco™) and incubated for 18 h in 37°C, 5% CO_2_ atmosphere.

### Optimized concentration of 1α,25(OH)_2_D_3_ to minimize A549 cytotoxicity and *B*. *pseudomallei* survival

To optimize the concentration of 1α,25(OH)_2_D_3_ for stimulation of A549 cells, the amount of lactate dehydrogenase enzyme (LDH) released was monitored using a method slightly modified from a previous study [25]. LDH release is an indication of plasma-membrane damage and thus of cytotoxicity. Briefly, A549 cells were plated in 24-well plates at a concentration of 10^5^ cells/well and incubated at 37°C, 5%, CO_2_ atmosphere overnight. Subsequently, the cells were treated with 1α,25(OH)_2_D_3_ (Sigma-Aldrich, St. Louis, MO) dissolved in ethanol (ETOH) (Sigma-Aldrich) at concentrations of 10^−8^, 10^−7^ and 10^−6^ M for 0, 12, 24 and 48 h. A549 cells with ETOH were used as vehicle control. Cell viability was measured using the LDH assay according to the manufacturer’s instructions (Sigma-Aldrich) at the optical density of 492 nm using a microplate reader (Sunrise™, TECAN, Switzerland). Cytotoxicity (%) was calculated as 100× [(experimental LDH release)—(control LDH release) / (maximum LDH release)—(control LDH release)], where values for LDH released from the control and maximum LDH release were obtained from the untreated normal cells and completely lysed cells (using 0.1% Triton X-100), respectively.

The effect of 1α,25(OH)_2_D_3_ on *B*. *pseudomallei* H777 survival was determined. One hundred micro liters of mid log-phase culture (10^6^ CFU/ml) was added directly into the wells in the presence of 10^−6^ M 1α,25(OH)_2_D_3_ or ETOH (vehicle control). Untreated and treated bacterial cultures were incubated for 0, 2, 4, 8, 12 h before bacterial viability was observed using the drop plate technique. The assay was performed on three separate occasions, with triplicate determinations each time.

### 1α,25(OH)_2_D_3_ pretreatment of A549 cells

A549 cells were plated in 24-well plates at a concentration of 10^5^ cells/well and incubated at 37°C, 5% CO_2_ atmosphere overnight. New complete medium containing 10^−6^ M of 1α,25(OH)_2_D_3_ was added to A549 cells and further incubated at 37°C, 5% CO_2_ atmosphere for 24 h. Cells receiving ETOH were used as the control. Next, cells were washed, and new complete medium was added before proceeding to further experiments.

### Effect of 1α,25(OH)_2_D_3_ -pretreatment on *B*. *pseudomallei* adhesion to A549 cells

The infection process was performed according to a previously described method with some modifications [26]. Cells from the mid-log phase of *B*. *pseudomallei* H777 culture (10^6^ CFU/ml) were added into the A549 cells to reach the optimal multiple of infection (MOI), approximately 1. Subsequently, the plate was gently shaken at 125 rpm on a shaking machine for 1 min and further incubated at 37°C, 5% CO_2_ atmosphere, for 2 h to allow bacterial adhesion.

The adhesion assay was performed as previously described with some modifications [26]. In brief, after co-culture of *B*. *pseudomallei* H777 at MOI of 1 with A549 cells pretreated with 10^−6^ M 1α,25(OH)_2_D_3_ for 2 h, the unbound bacteria were removed. The A549 cells were then washed three times using sterile phosphate buffer saline (PBS), pH 7.5 and then lysed with 1 ml of 0.1% Triton^®^ X-100 (MERCK, Burlington, MA) for 5 min. The lysate was vigorously mixed by pipetting, serially diluted, plated on LB agar by drop plate technique and incubated at 37°C for 48 h. The numbers of adhered bacteria were expressed as CFU and the percentage of bacterial adhesion calculated from three independent experiments, with triplicate determinations each time.

### Effect of 1α,25(OH)_2_D_3_-pretreatment on *B*. *pseudomallei* internalization into A549 cells

The internalization assay was performed as previously described [26]. In brief, after 2 h of bacterial adhesion, the culture medium containing extracellular bacteria was removed. The infected cells were then washed 3 times with PBS, pH 7.5. Medium containing 250 μg/ml kanamycin (KAN) (Sigma-Aldrich) was added and further incubated for 2 h to inhibit the growth of residual extracellular bacteria. After 4 h of incubation, the cells were washed and lysed as described above. The numbers of internalized bacteria in the lysate were quantified using the drop plate technique on LB agar. The percentage of bacteria that had internalized was calculated from three independent experiments, with triplicate determinations each time.

### Effect of 1α,25(OH)_2_D_3_-pretreatment on *B*. *pseudomallei* multiplication in A549 cells

For the bacterial multiplication assay, the numbers of intracellular bacteria were monitored further at 8 and 12 h post infection as previously described [26]. After elimination of the extracellular bacteria with KAN (250 μg/ml) for 2 h, the cells were then washed and incubated with culture medium containing 20 μg/ml of KAN for another 2 h to kill any remaining extracellular bacteria. Eight and 12 h after infection, the intracellular bacteria were harvested, serially diluted, and quantified using the drop plate technique on LB agar. The number of intracellular bacteria was calculated from three independent experiments, with triplicate determinations each time.

### Formation of multinucleated giant cells (MNGCs)

The 10^−6^ M 1α,25(OH)_2_D_3_ -pretreated A549 cells were infected with *B*. *pseudomallei* H777 as previously described [26]. Controls were ETOH-pretreated and untreated A549 cells exposed to infection with *B*. *pseudomallei* H777. At 0, 8, 10, and 12 h post infection, cells were collected and washed 3 times with PBS, pH 7.5, then fixed with methanol for 1 min. The fixed cells were stained with Giemsa dye (Sigma-Aldrich) and incubated for 3 min. The stained cells were washed with tap water and air dried at room temperature. The cells were then enumerated under a microscope at 400X magnification for MNGC formation [27]. Any large cells with a single cell membrane but more than three nuclei were recorded as an MNGCs. MNGC formation index was analyzed as 100 × (nuclei counts observed in MNGC) / (nuclei counts observed in MNGC + nuclei counts observed in normal cell). This assay was performed on three separate occasions, with triplicate determinations each time.

To confirm the formation of MNGCs, fluorescent cell staining was further performed. The 10^−6^ M 1α,25(OH)_2_D_3_ -pretreated, ETOH-pretreated and untreated A549 cells were infected with *B*. *pseudomallei* H777 as previously mentioned. Negative controls containing no *B*. *pseudomallei* were included. At 12 h post infection, cells were washed and fixed in 4% paraformaldehyde (Sigma-Aldrich) for 10 min and then permeabilized with 0.1% (v/v) Triton X-100 for 5 min [28]. The nucleus of cells was stained with 4′, 6-diamidino-2-phenylindole (DAPI) (Molecular Probes, Eugene, OR) according to manufacturer’s instructions. The cells were observed under fluorescence microscope (Nikon ECLIPSE Ti, Tokyo, Japan) at 100× and 200× magnification and imaging with camera attachment and imaging software (The Nikon NIS-Elements). This assay was performed on three separate occasions, with triplicate determinations each time.

### hCAP-18/LL-37 mRNA expression

New complete medium containing 10^−6^ M of 1α,25(OH)_2_D_3_ was added to an overnight culture of A549 cells (10^5^ cells/well) and further incubated at 37°C, 5% CO_2_ atmosphere, for 24 h. Cells with media and ETOH served as negative and vehicle controls, respectively. The cells were collected, and total RNA was extracted using the TRIzol reagent (Invitrogen, Carlsbad, CA). The cDNA was synthesized by reverse-transcription-polymerase chain reaction (RT-PCR) using M-MLV reverse transcriptase (Promega, Promega Corporation, WI) with oligo (dT) primers (Promega). PCR was performed using RBC Taq DNA polymerase (RBC Bioscience, Taipei, Taiwan) with specific primers for target genes. The mRNA expression was converted to a value relative to GAPDH mRNA expression and presented as fold increase relative to the results for medium alone. Oligonucleotide primers for PCR amplification of human cathelicidin gene (hCAP-18/LL-37) and GAPDH were designed from Homo sapiens cathelicidin antimicrobial peptide (CAMP) mRNA sequence (NM_004345.3) and Homo sapiens glyceraldehyde-3-phosphate dehydrogenase (GAPDH) mRNA sequence (NM_002046.3) available in Genbank by Primer 3 program. The primer sequences are hCAP-18/LL-37-F_CTAGAGGGAGGCAGACATGG and hCAP-18/LL-37-R_AGGAGGCGGTAGAGGTTAGC (for Homo sapiens cathelicidin antimicrobial peptide (*CAMP*) gene); GAPDH-F_GAGTCCACTGGCGTCTTCA and GAPDH- R_GGGGTGCTAAGCAGTTGGT (for the *GAPDH* gene).

### Cytokine detection

The 1α,25(OH)_2_D_3_-pretreated cells were infected with *B*. *pseudomallei* H777 at MOI of 1 at 37°C, 5% CO_2_ atmosphere for 12 h (a period of time that minimized MNGC formation). Untreated A549 cells in the presence or absence of *B*. *pseudomallei* H777 were used as controls. After infection, the A549 cells were washed three times with PBS, pH 7.5 and harvested using trypsin-EDTA solution (0.25% trypsin, 0.1% EDTA) (Corning™, Corning, NY). The cell suspension was centrifuged at 10,000 rpm for 5 min. After centrifugation, intracellular proteins were extracted using PRO-PREP™ Protein Extraction Solution (iNtRON Biotechnology, Gyeonggi, Korea) at -20°C for 20 min. The cytokines (CXCL1, CXCL12, IFN-γ, IL-1β, IL-2, IL-4, IL-6, IL-8, IL-10, IL-18, TNF-α, PAI-1 and MIF) were detected using Proteome Profiler™ Human Cytokine Array Kit (R&D Systems, Minneapolis, MN) according to the manufacturer’s instructions. Images were obtained using a chemiluminescence imaging system and the densitometry data were analyzed using ImageQuant TL Software version 8.1 (Amersham™ Imager 600, GE Healthcare Life Sciences, Chicago, IL). The assay was performed on two separate occasions, with duplicate determinations each time.

### Statistical analysis

Data are presented as mean ± standard deviation (SD). Statistical analyses of data from all experiments were done using T-test and one-way ANOVA by Tukey’s multiple comparisons test. All statistics were performed using GraphPad Prism software (version 9).

## Results

### The optimized 1α,25(OH)_2_D_3_ concentration on A549 cell cytotoxicity and *B*. *pseudomallei* cells

**Fig 1:**
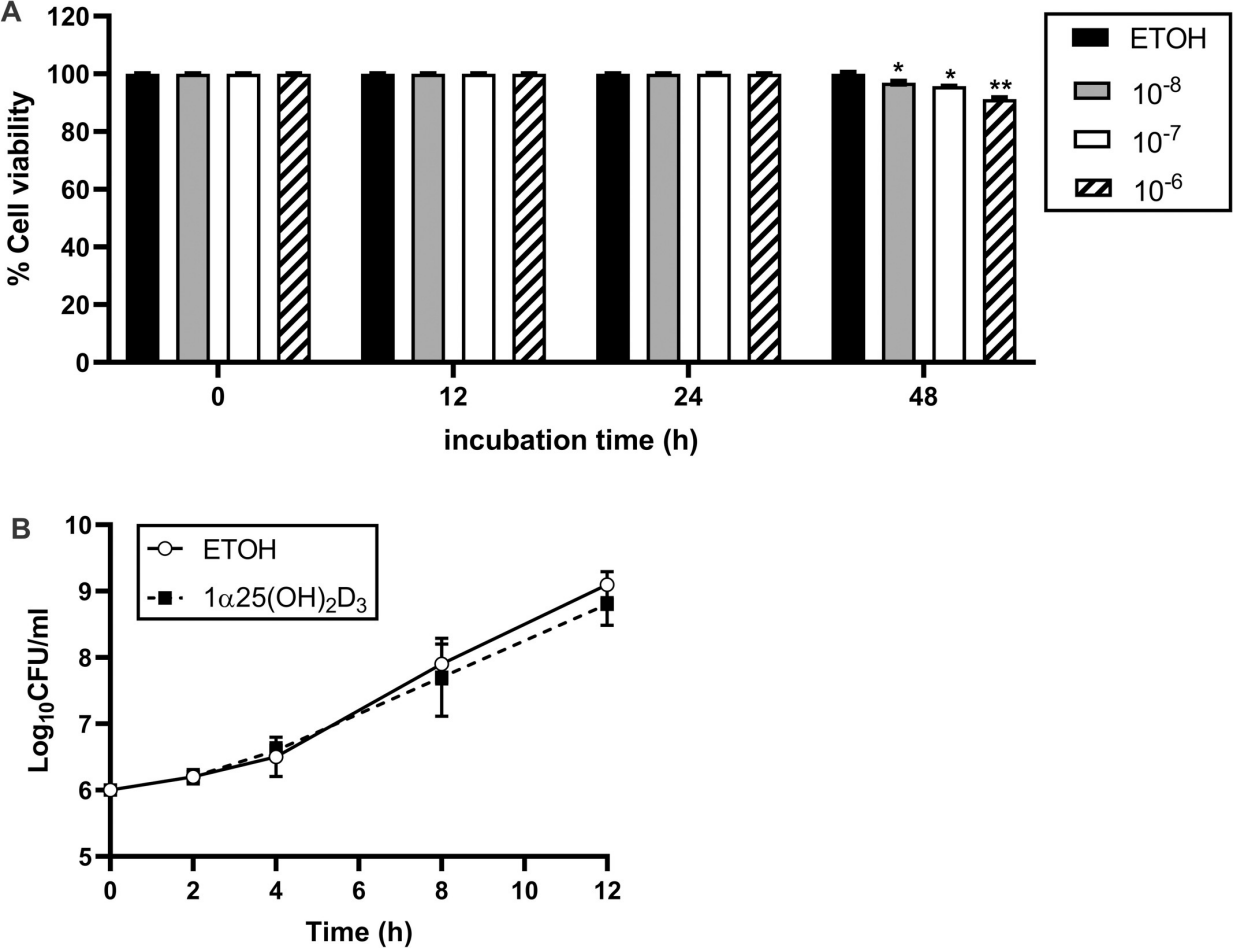
Effect of 1α,25(OH)_2_D_3_ on the survival of A549 cells and *B*. *pseudomallei* H777. (A) A549 cells were incubated with 1α,25(OH)_2_D_3_ (10^−8^ M, 10^−7^ M, 10^−6^ M) for 0, 12, 24, 48 h. Cytotoxicity was determined using the LDH assay. (B) *B*. *pseudomallei* H777 was incubated with 1α,25(OH)_2_D_3_ (10^−6^ M) for 0, 2, 4, 8 and 12 h. Bacterial viability was determined using the drop plate technique. The results are shown as mean ± SD from three independent experiments each carried out in triplicate. *P < 0.05 and **P < 0.01.

To optimize the level of 1α,25(OH)_2_D_3_ required for stimulation of A549 cells, the released LDH was measured in the supernatant after 24 h of 1α,25(OH)_2_D_3_ pretreatment. Cytotoxicity was not observed at concentrations of 1α,25(OH)_2_D_3_ (10^−8^, 10^−7^, 10^−6^ M) after 0, 12 or 24 h. Some cytotoxicity was apparent at 48 h post exposure with 10^−6^ M 1α,25(OH)_2_D_3_ (Fig 1A). Thus, the highest concentration of 1α,25(OH)_2_D_3_ (10^−6^ M) was selected for stimulation of A549 cells for 24 h before proceeding to bacterial infection.

The antimicrobial assay revealed that 1α,25(OH)_2_D_3_ alone did not exhibit antimicrobial effects against *B*. *pseudomallei* H777 within 12 h of infection (Fig 1B). Thus, 10^−6^ M of 1α,25(OH)_2_D_3_ was selected for stimulation of A549 cells in further investigations.

### 1α,25(OH)_2_D_3_-pretreated A549 cells reduced *B*. *pseudomallei* internalization

**Fig 2:**
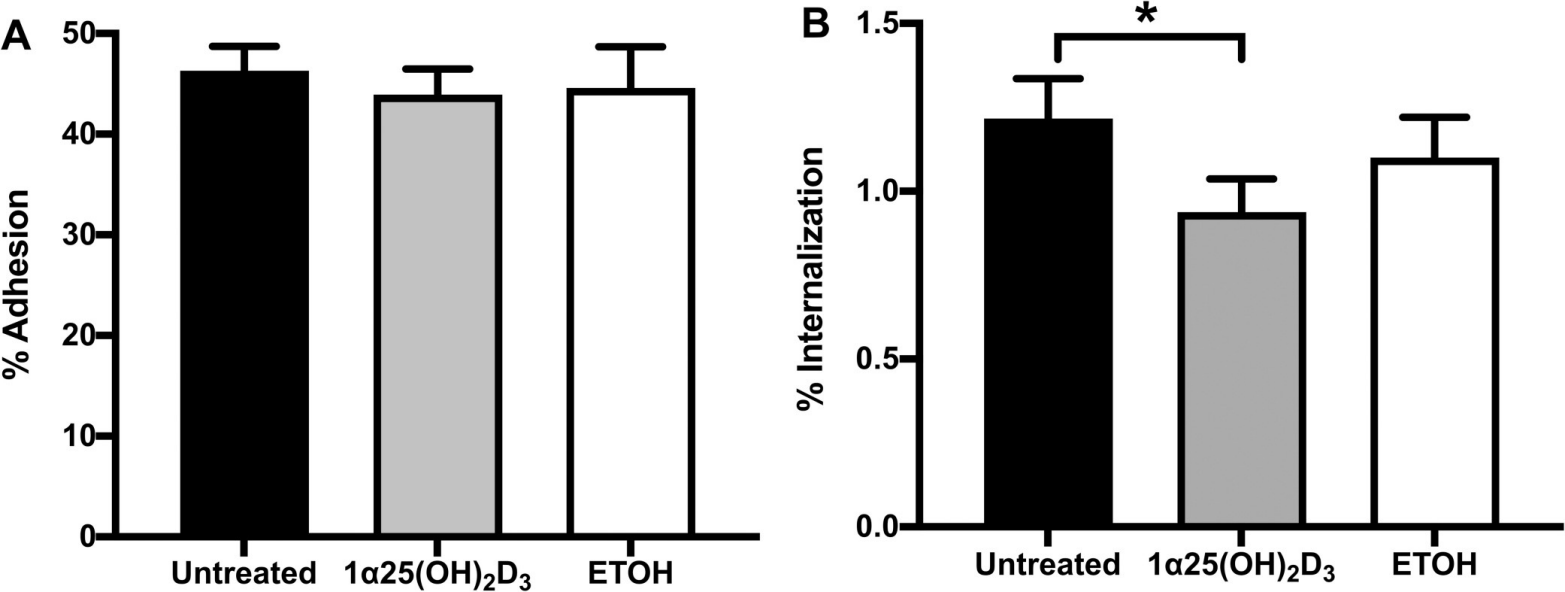
Effect of pretreatment of A549 cells with 1α,25(OH)_2_D_3_ on aspects of *B*. *pseudomallei* infection. The 1α,25(OH)_2_D_3_-pretreated A549 cells were incubated with *B*. *pseudomallei* H777. (A) Bacterial adhesion, and (B) internalization were determined using the plate count technique. The results are shown as mean ± SD from three independent experiments carried out in triplicate. *P < 0.05.

To determine whether 1α,25(OH)_2_D_3_ can minimize the capability of *B*. *pseudomallei* to adhere to human lung epithelial cells, the A549 cells were pre-exposed to 10^−6^ M 1α,25(OH)_2_D_3_ for 24 h followed by co-cultivation with *B*. *pseudomallei* H777 at MOI 1 (Fig 2). At 2 h post infection, the proportion of adhering bacteria was investigated. The percentage of *B*. *pseudomallei* adhering to cells pretreated with 1α,25(OH)_2_D_3_ was similar to that of the untreated control (Fig 2A). In contrast, after antibiotic protection assay and incubation for a further 2 h, the percentage of bacteria internalized in 1α,25(OH)_2_D_3_-pretreated cells was significantly lower than in untreated cells (*P* < 0.05) (Fig 2B). These results revealed the potential of 1α,25(OH)_2_D_3_ to interrupt the internalization ability of *B*. *pseudomallei* into the human lung epithelial cells.

### The expression of hCAP-18/LL-37 mRNA in 1α,25(OH)_2_D_3_-pretreated A549 cells

**Fig 3:**
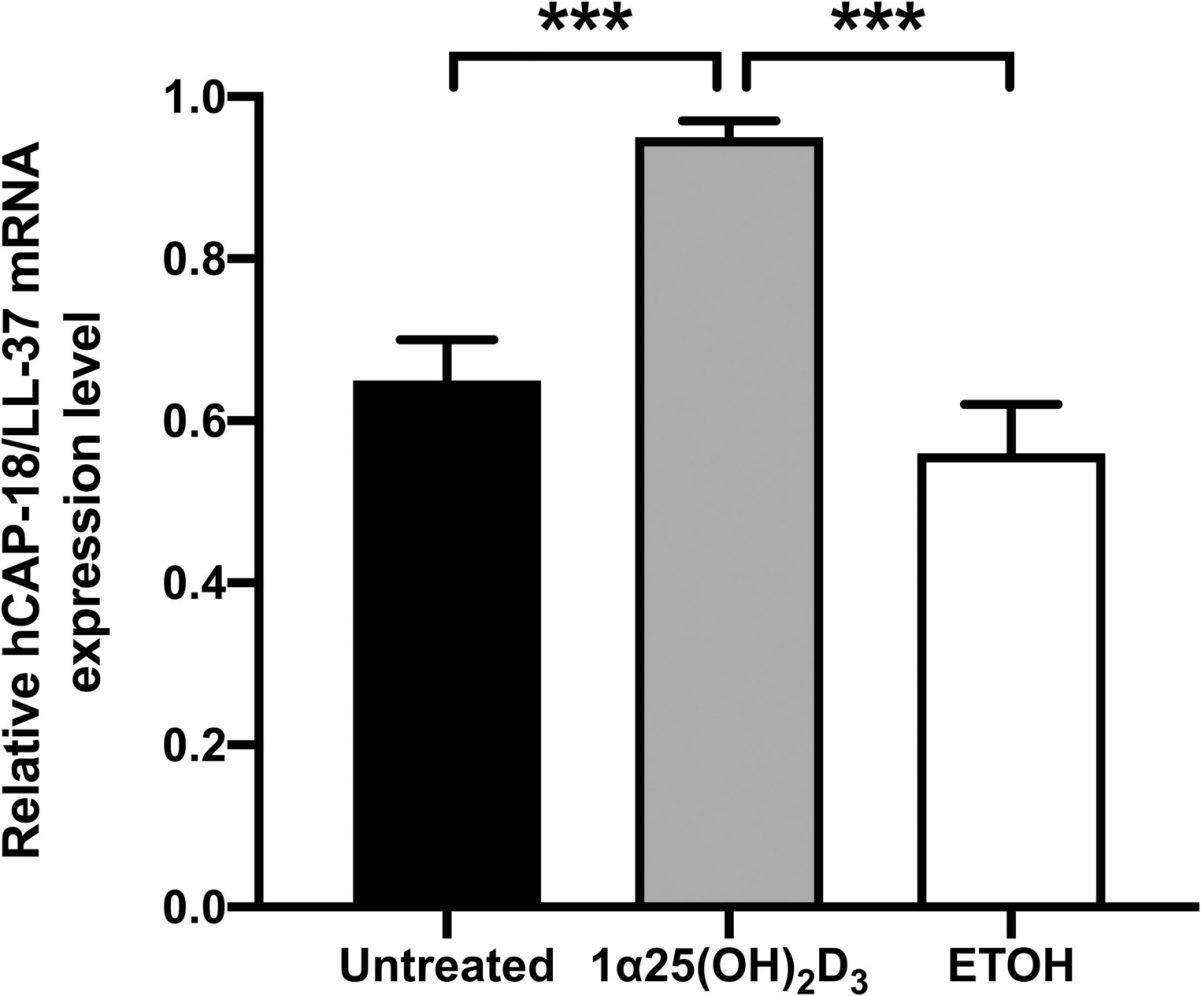
Expression of hCAP-18/LL-37 mRNA in 1α,25(OH)_2_D_3_-pretreated A549 cells. A549 cells were incubated with 10^−6^ M 1α,25(OH)_2_D_3_ for 24 h. The expression of target genes was determined using quantitative RT-PCR. The relative quantitation of each gene was normalized to GADPH. The results are shown as mean ± SD from three independent experiments carried out in triplicate and presented as relative ratio. ***P < 0.001.

Many studies have suggested a link between vitamin D treatment and an increased production of hCAP18/LL-37 protein [22, 29, 30]. We also investigated the effect of pretreatment with 1α,25(OH)_2_D_3_ on hCAP-18/LL-37 expression by A549 cells. We found that expression of hCAP-18/LL-37 mRNA in pretreated cells was significantly greater than in untreated and ETOH-treated cells (*P* < 0.001) (Fig 3). This result suggested that 1α,25(OH)_2_D_3_ can stimulate the expression of hCAP-18/LL-37 mRNA in A549 cells.

### Multiplication and formation of MNGCs by *B*. *pseudomallei* within 1α,25(OH)_2_D_3_-pretreated A549 cells

**Fig 4:**
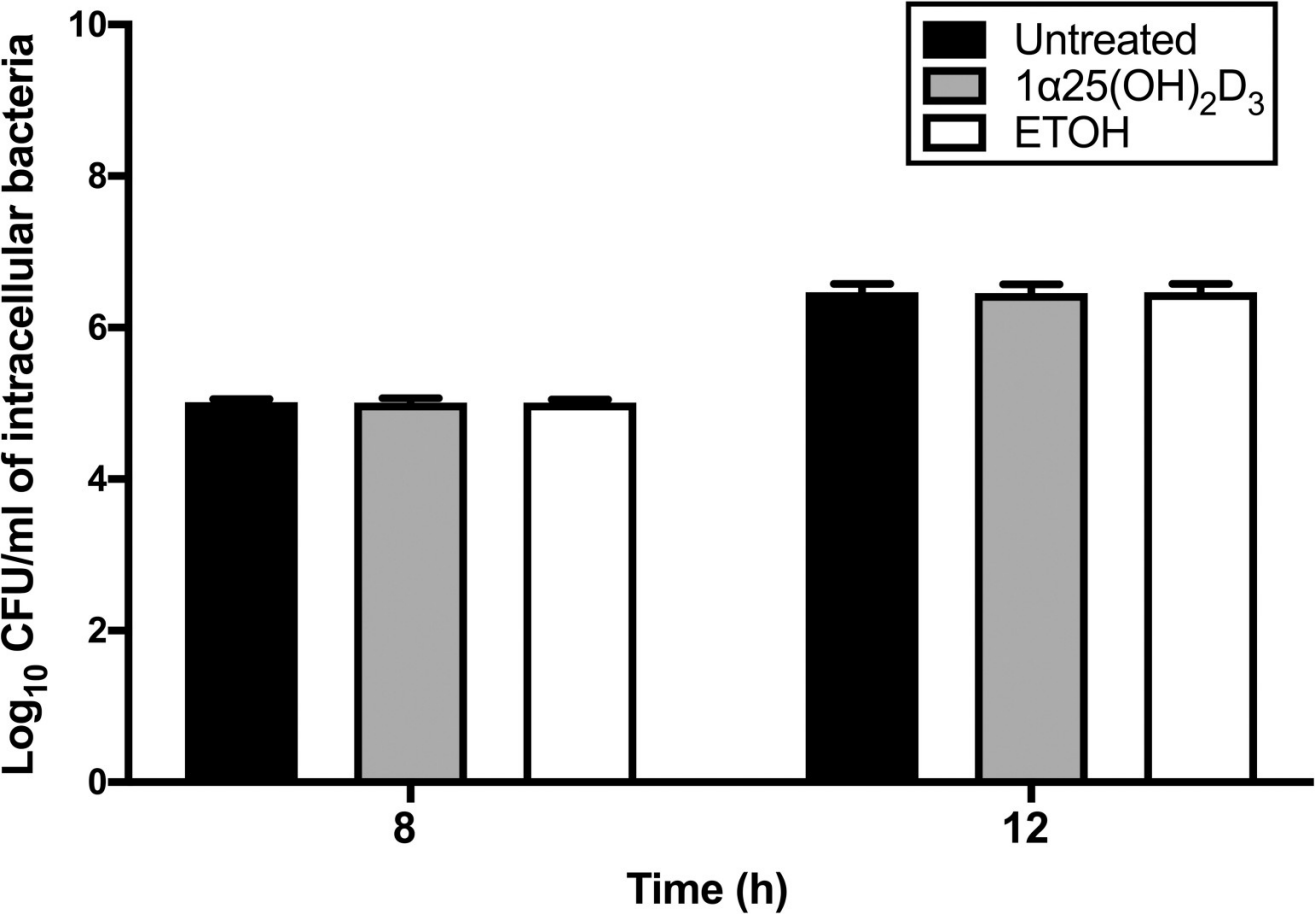
Effect of 1α,25(OH)_2_D_3_ pretreatment of A549 cells on *B*. *pseudomallei* multiplication. The 1α,25(OH)_2_D_3_-pretreated A549 cells were incubated with *B*. *pseudomallei* H777. Bacterial multiplication was determined using the plate count technique. The results are shown as mean ± SD from three independent experiments carried out in triplicate.

**Fig 5:**
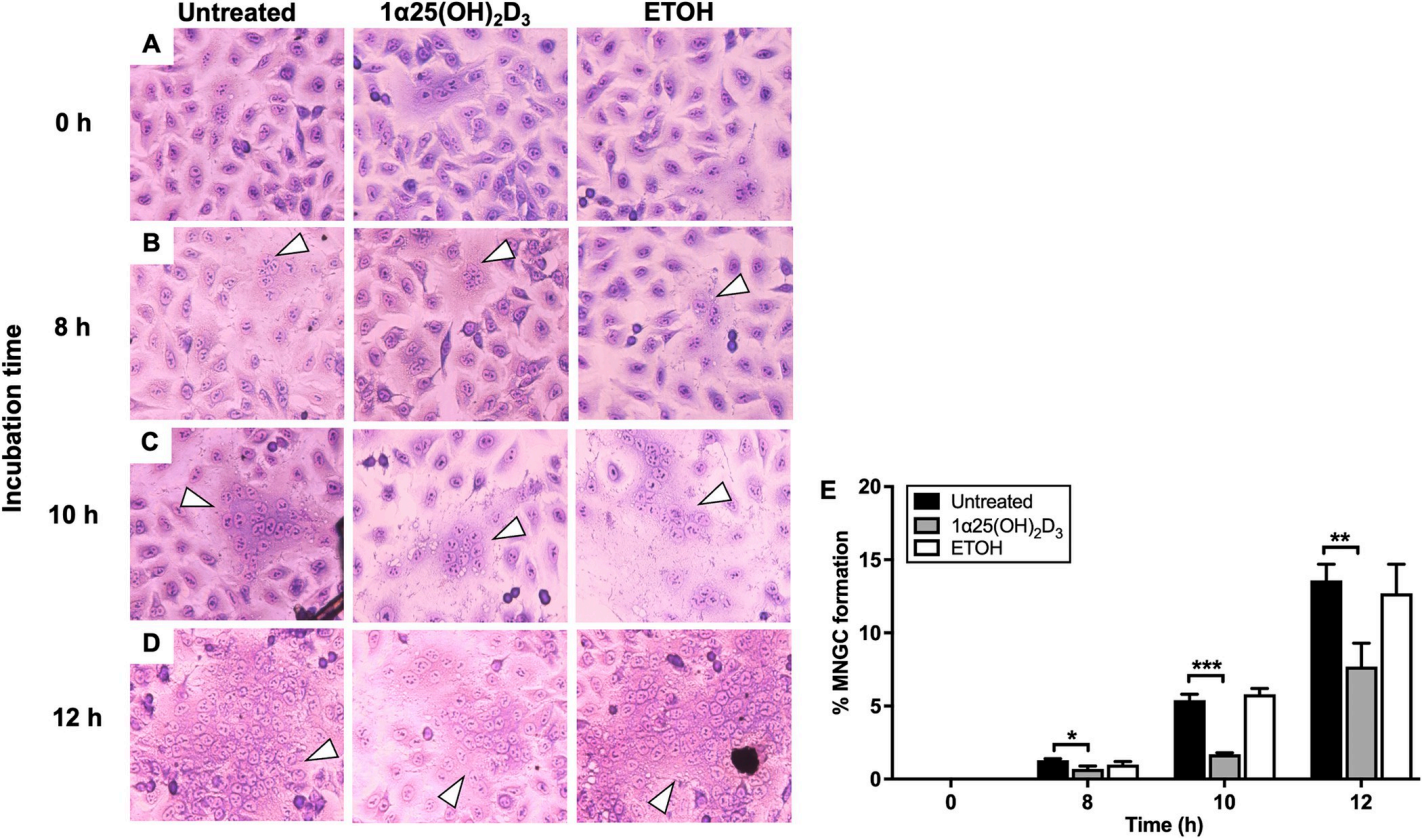
MNGC formation is lower in 1α,25(OH)_2_D_3_-pretreated A549 cells during *B*. *pseudomallei* infection. Untreated, 1α,25(OH)_2_D_3_-pretreated and ETOH-pretreated A549 cells were infected with *B*. *pseudomallei*. MNGC formation in each condition was determined at 0 (A), 8 (B), 10 (C) and 12 h (D) and expressed as percentages (E). The white arrows indicate the formation of MNGCs. The results are shown as mean ± SD from three independent experiments carried out in triplicate. *P < 0.05, **P < 0.01 and ***P < 0.001.

The impact of 1α,25(OH)_2_D_3_ pretreatment of A549 cells on *B*. *pseudomallei* multiplication was observed 8 and 12 h post-infection. The numbers of *B*. *pseudomallei* H777 bacilli within pretreated and untreated A549 cells were similar (Fig 4). Furthermore, we estimated the extent of MNGC formation by Giemsa staining of pretreated and untreated cells in the presence of *B*. *pseudomallei* at 0, 8, 10 and 12 h (Fig 5). Interestingly, MNGC formation among the 1α,25(OH)_2_D_3_-pretreated cells was markedly reduced relative to controls at 8, 10 and 12 h (Fig 5B–5D). MNGC formation in untreated and ETOH-pretreated A549 cells (vehicle control) increased in a time-dependent manner within 12 h of *B*. *pseudomallei* infection. The percentage of MNGCs formed among the 1α,25(OH)_2_D_3_ pretreated cells was significantly lower than controls at 8 h (*P* < 0.05), 10 h (*P* < 0.001) and 12 h (*P* < 0.01) (Fig 5E).

**Fig 6:**
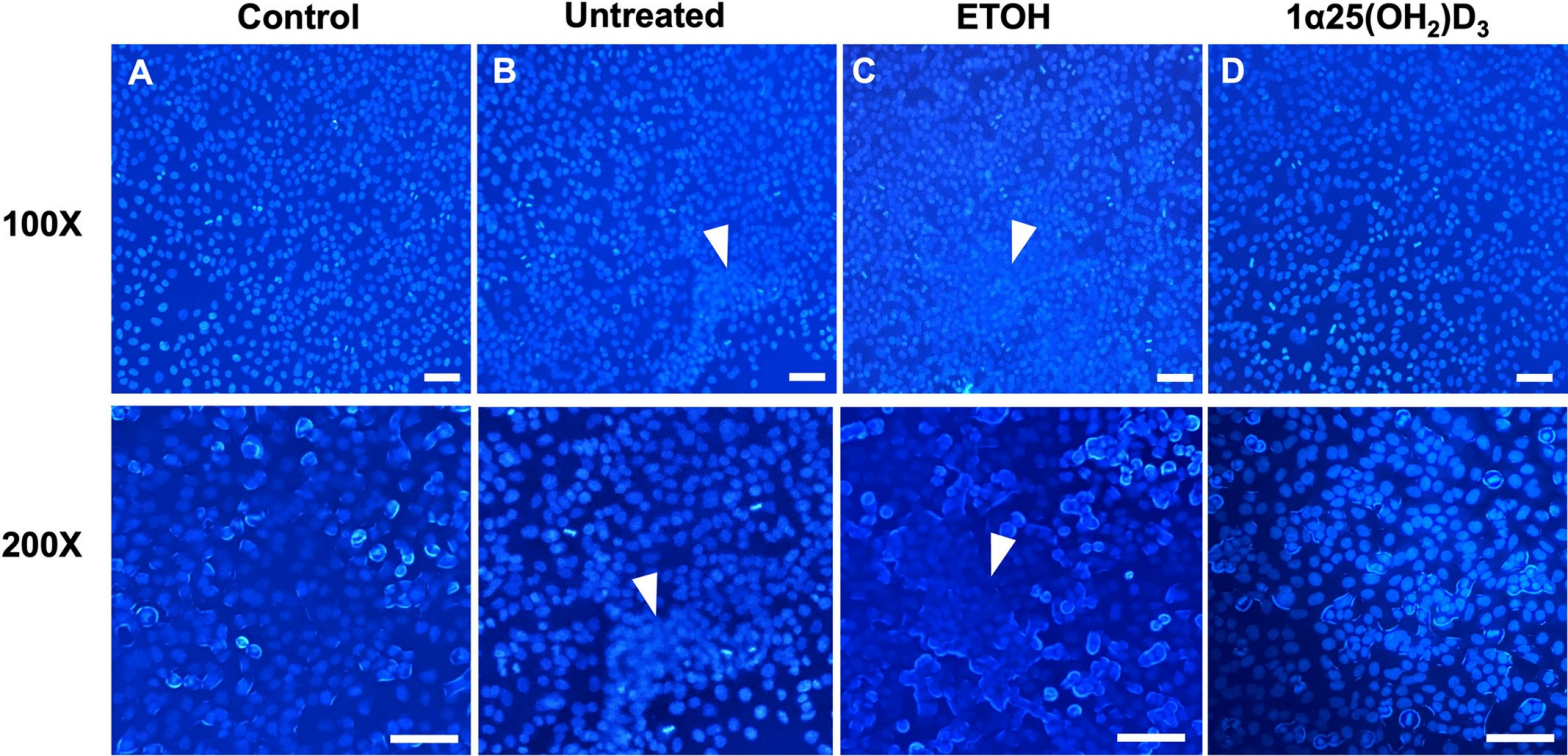
Confirmation of MNGC reduction in 1α,25(OH)_2_D_3_-pretreated A549 cells during *B*. *pseudomallei* infection. A549 cells without *B*. *pseudomallei* were used as a negative control (A). Untreated (B), ETOH-pretreated (C) and 1α,25(OH)_2_D_3_-pretreated (D) A549 cells were infected with *B*. *pseudomallei* at MOI 1 for 12 h. Cells were then fixed and stained with DAPI (blue). The white arrows indicate the formation of MNGCs. Scale bar = 100 μm.

MNGC reduction of the 1α,25(OH)_2_D_3_-pretreated A549 cells during *B*. *pseudomallei* infection was confirmed using nuclei staining (DAPI) as shown in Fig 6. Exposure of untreated and ETOH-pretreated A549 cells to *B*. *pseudomallei* for 12 h resulted in the development of MNGCs through the cell–cell fusion (Fig 6B and 6C). However, similar morphology of 1α,25(OH)_2_D_3_-pretreated A549 cells (Fig 6D) as control was observed (Fig 6A). These results indicated a potential of 1α,25(OH)_2_D_3_ to reduce MNGC formation, a pathogenesis marker of *B*. *pseudomallei* infection.

### Cytokine expression by 1α,25(OH)_2_D_3_-pretreated cells during *B*. *pseudomallei* infection

**Fig 7:**
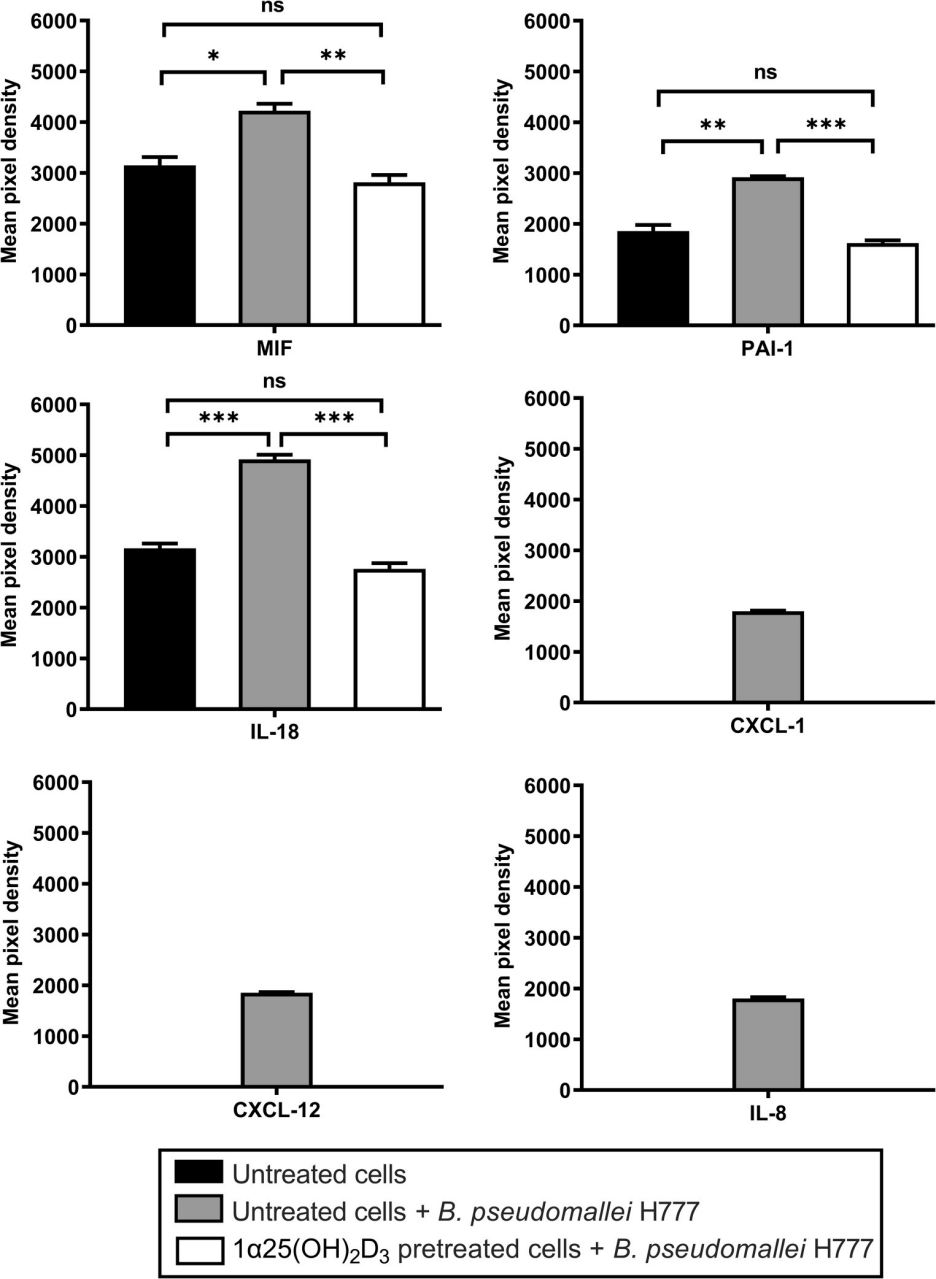
Cytokine expression during *B*. *pseudomallei* infection. Cytokine expression levels in all tested conditions were determined using cytokine antibody assays. The pixel density of each identified cytokine was calculated using ImageQuant TL Software. The results are shown as mean ± SD from two independent experiments carried out in duplicate. ns = not significant, *P < 0.05, **P < 0.01 and ***P < 0.001.

The competence of 1α,25(OH)_2_D_3_ to modulate inflammatory host-cell response via NF-κB was determined. The results demonstrated that untreated A549 cells infected with *B*. *pseudomallei* expressed significantly higher amounts of MIF, PAI-1, IL-18, CXCL1, CXCL12 and IL-8 when compared with uninfected cells at 12 h post-infection (Fig 7). Interestingly, the pretreated A549 cells infected with *B*. *pseudomallei* expressed significantly less MIF (*P* < 0.01), PAI-1 (*P* < 0.001) and IL-18 (*P* < 0.001) than did the untreated *B*. *pseudomallei*-infected cells. In addition, CXCL1, CXCL12 and IL-8 were completely downregulated in 1α,25(OH)_2_D_3_-pretreated infected cells. These results indicated that pretreatment of cells with 1α,25(OH)_2_D_3_ might reduce the production of pro-inflammatory cytokines during *B*. *pseudomallei* infection.

## Discussion

Melioidosis is a life-threatening disease in humans and is caused by *B*. *pseudomallei*. This pathogen can evade the host immune system due to its intracellular lifestyle, resulting in severe infections manifested as pneumonia and sepsis [10]. For that reason, a novel strategy to prevent or clear the initial steps of infection as well as reduce the pathogenesis of *B*. *pseudomallei* infection may provide many benefits. Vitamin D is a well-known essential micronutrient that has ability to modulate host immune responses by regulating gene expression in multiple signaling pathways, subsequently limiting microbial pathogenesis [14]. Despite multiple studies on other diseases, studies evaluating the potential of vitamin-D in melioidosis are lacking. In this study, the effect of an active metabolite of vitamin D_3_ (1α,25(OH)_2_D_3_) on each step of *B*. *pseudomallei* pathogenesis was investigated using a human lung epithelial cell line (A549). Active vitamin D can regulate the immune system by interacting with VDR to increase expression of vitamin D-regulated genes. Human lung epithelial cells have also been reported the expression of VDR in the apical layer but not in basal cells or epithelial stem cells [31]. The lung epithelium is the primary target of pulmonary infection with *B*. *pseudomallei* [32]. Thus we might expect to see vitamin D-mediated effects in an epithelial cell line *in vitro*. The concentration range of 1α,25(OH)_2_D_3_ for stimulation of lung epithelial cell lines was selected based on the concentration previously determined to be optimal for the stimulation of THP-1 cells [25]. This result confirmed the safety of the highest concentration used of 1α,25(OH)_2_D_3_ (10^−6^ M) on A549 cell lines.

Adhesion and invasion are the critical steps of *B*. *pseudomallei* pathogenesis. Our results showed that no interference with bacterial adhesion onto 1α,25(OH)_2_D_3_-pretreated cells was observed while the percentage of *B*. *pseudomallei* bacilli that internalized after 4 h was significantly reduced in pretreated cells. These findings demonstrate a protective role for vitamin-D in epithelial cells during *B*. *pseudomallei* invasion. This is in good agreement with a previous study that reported the pretreatment of HeLa cells with 1α,25(OH)_2_D_3_ for 24 h decreased the infectivity of *Chlamydia muridarum* [33]. Moreover, 1α,25(OH)_2_D_3_ could also attenuate colonization and invasion of adherent-invasive *Escherichia coli* (AIEC) on Caco-2-bbe monolayers [34]. Nevertheless, reduced bacterial multiplication was not observed in 1α,25(OH)_2_D_3-_pretreated A549 cells at 8 and 12 h post infection in this study. On the contrary, pretreatment of 1α,25(OH)_2_D_3_ can mediate the protective response of HeLa cells, resulting in dramatically inhibited intracellular replication of *C*. *muridarum* [33]. Moreover, 1α,25(OH)_2_D_3_ inhibits *M*. *tuberculosis* multiplication in human monocytes by the expression of human cathelicidin peptide (hCAP18/LL-37) which activates autophagy and induces antimycobacterial activity [35]. As previously stated, stimulation with 1α,25(OH)_2_D_3_ enhances the promoter of the hCAP18 gene which contains multiple VDR-response elements in keratinocytes, monocytes and neutrophils, leading to the induction of hCAP18 expression [36]. We also determined the expression of hCAP-18/LL-37 mRNA in 1α,25(OH)_2_D_3-_pretreated A549 cells and found significantly increased expression of hCAP-18/LL-37 mRNA at 24 h, consistent with previous results. This may explain the reduction of the *B*. *pseudomallei* internalization to A549 cells within the first 4 h of infection. However, *B*. *pseudomallei* can escape the phagosome utilizing the Type III Secretion System (TTSS) [37] and avoid host cell autophagy clearance during the late stages of infection [38]. Thus, the intracellular lifestyle and immune-evasion strategies of *B*. *pseudomallei* may strongly influence the outcome of 1α,25(OH)_2_D_3_ treatment and mediate the protective response of lung epithelial cells against the initial steps of bacterial infection.

As previously mentioned, internalized *B*. *pseudomallei* can induce cell-to-cell fusion, resulting in MNGC formation in both *in vitro* [9] and *in vivo* [39] models. The physiological role of *B*. *pseudomallei*-induced MNGC formation in pathogen survival remains to be elucidated. Several studies have suggested that MNGC formation enables *B*. *pseudomallei* to evade host immune surveillance or antibiotics [9] and spread to target organs [13]. Interestingly, we found that MNGC formation was significantly reduced after infection with *B*. *pseudomallei* H777 in 1α,25(OH)_2_D_3_-pretreated cells at 8, 10 and 12 h post infection without any effect on intracellular bacterial numbers. The mechanism of MNGC formation appears to remain poorly understood. Recent studies have mentioned the role of bacterial Type IV secretion system (T6SS) in this process [11, 40]. However, the host cellular factor that is targeted by the T6SS to mediate MNGC formation is as yet unknown. Sangsri et al. [41] demonstrated that infection of *B*. *pseudomallei* in A549 cells (tetraspanin CD81 gene knockout) or A549 cells treated with anti-CD81, led to significant inhibition of MNGC formation without any effect on the number of intracellular *B*. *pseudomallei*. Tetraspanin CD81 belongs to a superfamily of transmembrane proteins which displays properties such as cell adhesion, motility and proliferation [42]. Interestingly, CD81-expressing cells are potential targets for 1α,25(OH)_2_D_3_ to regulate proliferation and cytokine production [43, 44]. However, the effect of 1α,25(OH)_2_D_3_ on CD81 expression or function has never been reported in A549 cells. The current study is the first to provide direct evidence that 1α,25(OH)_2_D_3_ can mediate host epithelial-cell responses to *B*. *pseudomallei* internalization and MNGC formation.

Previous studies suggest that infection with *B*. *pseudomallei* also induces the secretion of pro-inflammatory cytokines both *in vitro* and *in vivo* [32]. Therefore, we determined cytokine expression profiles in 1α,25(OH)_2_D_3_-pretreated A549 cells infected with *B*. *pseudomallei*. Expression levels of several cytokines and chemokines were elevated in untreated cells with *B*. *pseudomallei* including MIF, PAI-1, IL-18, IL-8, CXCL1 and CXCL12. MIF has emerged as an important effector molecule for innate immunity in severe sepsis, e.g., melioidosis. A high level of MIF has been associated with poor outcomes in melioidosis patients [45]. Moreover, elevated expression of PAI-1, a regulator of inflammation and fibrinolysis, has been linked to poor clinical outcomes in severe melioidosis cases [46]. A pro-inflammatory chemokine IL-8 was generally induced by *B*. *pseudomallei* infection in cultured epithelial cells [26] and was also observed to be elevated in melioidosis patients compared with uninfected individuals [47]. This cytokine may have a protective role as a pivotal mediator in the early antimicrobial host response in *B*. *pseudomallei*-infected mice [48, 49]. Nevertheless, high levels of both IL-8 and IL-18 have been reported in serum of patients with severe melioidosis [50, 51]. Increased inflammatory-cell recruitment to the lungs by CXCL1 has a detrimental effect during melioidosis [52]. However, CXCL12, a lymphocyte chemoattractant, has never been reported in melioidosis. Interestingly, pretreatment of A549 cells with 1α,25(OH)_2_D_3_ led to statistically significant reduction in the cytokine-mediated host response (MIF, PAI-1 and IL-18) to levels comparable to those of the normal condition. CXCL1, CXCL12 and IL-8 could not be detected in 1α,25(OH)_2_D_3_-pretreated cells during *B*. *pseudomallei* infection. The mechanism by which 1α,25(OH)_2_D_3_ is able to reduce inflammatory response in infectious disease is not fully understood. Our results indicated that pretreatment of cultured epithelial cells with 1α,25(OH)_2_D_3_ has the potential to reduce inflammation during *B*. *pseudomallei* infection which may be further used to decrease the excessive inflammatory response in melioidosis patients.

## Conclusion

In summary, our studies have revealed that pretreatment of A549 cells with 1α,25(OH)_2_D_3_ can upregulate the expression of hCAP-18/LL-37 which may contribute to the reduction of *B*. *pseudomallei* internalization in these cells. Moreover, *B*. *pseudomallei*-induced MNGC formation was distinctly decreased in 1α,25(OH)_2_D_3_-pretreated A549 cells. There was also a reduction in the production of excessive pro-inflammatory cytokines. These data suggest the potential use of 1α,25(OH)_2_D_3_ as an adjunctive therapy for melioidosis patients.

## Supporting information

S1 Table The densitometry data of cytokine expression levels. (DOCX)

S2 Table The minimal data set underlying the results. (DOCX)
