# Supplementary material for: Vitamin D (1α,25(OH)2D3) supplementation minimized multinucleated giant cells formation and inflammatory response during Burkholderia pseudomallei infection in human lung epithelial cells
Source: PLoS One. 2023 Feb 9;18(2):e0280944. doi: 10.1371/journal.pone.0280944 (PMC9910702; doi:10.1371/journal.pone.0280944)
Supplement: S1 Table — (DOCX) [file pone.0280944.s001.docx]

**S1 Table. The densitometry data of cytokine expression levels.**

| **Cytokine** | **Pixel density (Mean *±* SD)** | | |
| --- | --- | --- | --- |
|  | **Untreated** | **Untreated + *B. pseudomallei*** | **1α,25(OH)_2_D_3_ pretreated cells + *B. pseudomallei*** |
| CXCL1 | N/D | 1,798.5 ± 14.9 | N/D |
| CXCL12 | N/D | 1,854.5 ± 10.6 | N/D |
| IFN-γ | N/D | N/D | N/D |
| IL-1β | N/D | N/D | N/D |
| IL-2 | N/D | N/D | N/D |
| IL-4 | N/D | N/D | N/D |
| IL-6 | N/D | N/D | N/D |
| IL-8 | N/D | 1,803 ± 25.5 | N/D |
| IL-10 | N/D | N/A | N/D |
| IL-18 | 3,168 ± 93.3 | 4,918.5 ± 89.8 | 2,765 ± 111.7 |
| MIF | 3,149 ± 162.6 | 4,223 ± 138.6 | 2,811.5 ± 145 |
| PAI-1 | 1,856.5 ± 120.9 | 2,919 ± 18.4 | 1,620 ± 54.5 |
| TNF-α | N/D | N/D | N/D |

N/D = Not detected.
