# Supplementary material for: Vitamin D (1α,25(OH)2D3) supplementation minimized multinucleated giant cells formation and inflammatory response during Burkholderia pseudomallei infection in human lung epithelial cells
Source: PLoS One. 2023 Feb 9;18(2):e0280944. doi: 10.1371/journal.pone.0280944 (PMC9910702; doi:10.1371/journal.pone.0280944)
Supplement: S2 Table — (DOCX) [file pone.0280944.s002.docx]

**S2 Table. The minimal data set underlying the results.**

| **Fig** | **Mean** | **S.D.** | **Statistical method used** | **P value** | **Samples** |
| --- | --- | --- | --- | --- | --- |
| **Fig. 1A** (Cytotoxicity: 0 h) | | | One-way ANOVA with Tukey's multiple comparisons test | - |  |
| ETOH | 100 | 0.02 |  |  | 3 |
| 10^-8^ | 100 | 0.02 |  |  | 3 |
| 10^-7^ | 100 | 0.03 |  |  | 3 |
| 10^-6^ | 100 | 0.03 |  |  | 3 |
| **Fig. 1A** (Cytotoxicity: 12 h) | | | One-way ANOVA with Tukey's multiple comparisons test | - |  |
| ETOH | 100 | 0.05 |  |  | 3 |
| 10^-8^ | 100 | 0.05 |  |  | 3 |
| 10^-7^ | 100 | 0.01 |  |  | 3 |
| 10^-6^ | 100 | 0.06 |  |  | 3 |
| **Fig. 1A** (Cytotoxicity: 24 h) | | | One-way ANOVA with Tukey's multiple comparisons test | - |  |
| ETOH | 100 | 0.04 |  |  | 3 |
| 10^-8^ | 100 | 0.04 |  |  | 3 |
| 10^-7^ | 100 | 0.16 |  |  | 3 |
| 10^-6^ | 100 | 0.01 |  |  | 3 |
| **Fig. 1A** (Cytotoxicity: 48 h) | | | One-way ANOVA with Tukey's multiple comparisons test | **P*<0.05  ***P*<0.01 |  |
| ETOH | 100 | 0.5 |  |  | 3 |
| 10^-8^ | 97.1 | 0.5 |  |  | 3 |
| 10^-7^ | 95.7 | 0.03 |  |  | 3 |
| 10^-6^ | 91.3 | 0.5 |  |  | 3 |
| **Fig. 1B** (*B. pseudomallei* survival in the presence of 1α,25(OH)_2_D_3_) | | |  |  |  |
| **0 h** | | | T-Test | **-** |  |
| ETOH | 6 | 0 |  |  | 6 |
| 1α,25(OH)_2_D_3_ | 6 | 0.1 |  |  | 6 |
| **2 h** | | | T-Test | **-** |  |
| ETOH | 6.2 | 0.1 |  |  | 6 |
| 1α,25(OH)_2_D_3_ | 6.2 | 0.1 |  |  | 6 |

| **Fig** | **Mean** | **S.D.** | **Statistical method used** | **P value** | **Samples** |
| --- | --- | --- | --- | --- | --- |
| **4 h** | | | T-Test | **-** |  |
| ETOH | 6.5 | 0.3 |  |  | 6 |
| 1α,25(OH)_2_D_3_ | 6.6 | 0.1 |  |  | 6 |
| **8 h** | | | T-Test | **-** |  |
| ETOH | 7.9 | 0.3 |  |  | 6 |
| 1α,25(OH)_2_D_3_ | 7.7 | 0.6 |  |  | 6 |
| **12 h** | | | T-Test | **-** |  |
| ETOH | 9.1 | 0.2 |  |  | 6 |
| 1α,25(OH)_2_D_3_ | 8.8 | 0.3 |  |  | 6 |
| **Fig. 2A** (Adhesion) | | | One-way ANOVA with Tukey's multiple comparisons test | - |  |
| Untreated | 46.3 | 2.4 |  |  | 9 |
| 1α,25(OH)_2_D_3_ | 43.9 | 2.5 |  |  | 9 |
| ETOH | 44.6 | 4.1 |  |  | 9 |
| **Fig. 2B** (Internalization) | | | One-way ANOVA with Tukey's multiple comparisons test | **P*<0.05 |  |
| Untreated | 1.2 | 0.1 |  |  | 9 |
| 1α,25(OH)_2_D_3_ | 0.9 | 0.1 |  |  | 9 |
| ETOH | 1.1 | 0.1 |  |  | 9 |
| **Fig. 3** (hCAP-18/LL-37 mRNA expression) | | | One-way ANOVA with Tukey's multiple comparisons test | ****P*<0.001 |  |
| Untreated | 0.6 | 0.05 |  |  | 6 |
| 1α,25(OH)_2_D_3_ | 0.9 | 0.02 |  |  | 6 |
| ETOH | 0.6 | 0.06 |  |  | 6 |
| **Fig. 4** (Intracellular bacteria: 8 h) | | | One-way ANOVA with Tukey's multiple comparisons test | - |  |
| Untreated | 5 | 0.04 |  |  | 9 |
| 1α,25(OH)_2_D_3_ | 5 | 0.06 |  |  | 9 |
| ETOH | 5 | 0.04 |  |  | 9 |
| **Fig. 4** (Intracellular bacteria: 12 h) | | | One-way ANOVA with Tukey's multiple comparisons test | - |  |
| Untreated | 6.5 | 0.11 |  |  | 9 |
| 1α,25(OH)_2_D_3_ | 6.4 | 0.11 |  |  | 9 |
| ETOH | 6.5 | 0.11 |  |  | 9 |

| **Fig** | **Mean** | **S.D.** | **Statistical method used** | **P value** | **Samples** |
| --- | --- | --- | --- | --- | --- |
| **Fig. 5E** (% MNGC formation: 0 h) | | | One-way ANOVA with Tukey's multiple comparisons test | - |  |
| Untreated | 0 | 0 |  |  | 6 |
| 1α,25(OH)_2_D_3_ | 0 | 0 |  |  | 6 |
| ETOH | 0 | 0 |  |  | 6 |
| **Fig. 5E** (% MNGC formation: 8 h) | | | One-way ANOVA with Tukey's multiple comparisons test | **P*<0.05 |  |
| Untreated | 1.3 | 0.1 |  |  | 6 |
| 1α,25(OH)_2_D_3_ | 0.7 | 0.2 |  |  | 6 |
| ETOH | 1 | 0.2 |  |  | 6 |
| **Fig. 5E** (% MNGC formation: 10 h) | | | One-way ANOVA with Tukey's multiple comparisons test | ****P*<0.001 |  |
| Untreated | 5.4 | 0.4 |  |  | 6 |
| 1α,25(OH)_2_D_3_ | 1.7 | 0.1 |  |  | 6 |
| ETOH | 5.8 | 0.4 |  |  | 6 |
| **Fig. 5E** (% MNGC formation: 12 h) | | | One-way ANOVA with Tukey's multiple comparisons test | ***P*<0.01 |  |
| Untreated | 13.6 | 1.1 |  |  | 6 |
| 1α,25(OH)_2_D_3_ | 7.7 | 0.1 |  |  | 6 |
| ETOH | 12.7 | 2 |  |  | 6 |
| **Fig. 7** (MIF) | | |  |  |  |
| Untreated | 3,149 | 162.6 | One-way ANOVA with Tukey's multiple comparisons test | **P*<0.05  ***P*<0.01 | 4 |
| Untreated + *B. pseudomallei* | 4,223 | 138.6 |  |  | 4 |
| 1α,25(OH)_2_D_3_ pretreated cells + *B. pseudomallei* | 2,811.5 | 145 |  |  | 4 |
| **Fig. 7** (PAI-1) | | |  |  |  |
| Untreated | 1,856.5 | 120.9 | One-way ANOVA with Tukey's multiple comparisons test | ***P*<0.01  ****P*<0.001 | 4 |
| Untreated + *B. pseudomallei* | 2,919 | 18.4 |  |  | 4 |
| 1α,25(OH)_2_D_3_ pretreated cells + *B. pseudomallei* | 1,620 | 54.5 |  |  | 4 |

| **Fig** | **Mean** | **S.D.** | **Statistical method used** | **P value** | **Samples** |
| --- | --- | --- | --- | --- | --- |
| **Fig. 7** (IL-18) | | | One-way ANOVA with Tukey's multiple comparisons test | ****P*<0.001 |  |
| Untreated | 3,168 | 93.3 |  |  | 4 |
| Untreated + *B. pseudomallei* | 4,918.5 | 89.8 |  |  | 4 |
| 1α,25(OH)_2_D_3_ pretreated cells + *B. pseudomallei* | 2,765 | 111.7 |  |  | 4 |
| **Fig. 7** (CXCL-1) | | | One-way ANOVA with Tukey's multiple comparisons test | ****P*<0.001 |  |
| Untreated | 0 | 0 |  |  | 4 |
| Untreated + *B. pseudomallei* | 1,798.5 | 14.9 |  |  | 4 |
| 1α,25(OH)_2_D_3_ pretreated cells + *B. pseudomallei* | 0 | 0 |  |  | 4 |
| **Fig. 7** (CXCL-12) | | |  |  |  |
| Untreated | 0 | 0 | One-way ANOVA with Tukey's multiple comparisons test | ****P*<0.001 | 4 |
| Untreated + *B. pseudomallei* | 1,854.5 | 10.6 |  |  | 4 |
| 1α,25(OH)_2_D_3_ pretreated cells + *B. pseudomallei* | 0 | 0 |  |  | 4 |
| **Fig. 7** (IL-8) | | |  |  |  |
| Untreated | 0 | 0 | One-way ANOVA with Tukey's multiple comparisons test | ****P*<0.001 | 4 |
| Untreated + *B. pseudomallei* | 1,803 | 25.5 |  |  | 4 |
| 1α,25(OH)_2_D_3_ pretreated cells + *B. pseudomallei* | 0 | 0 |  |  | 4 |
